# Bibliographical Notices

**Published:** 1838-05-23

**Authors:** 


### BIBLIOGRAPHICAL NOTICES.

POPULAR MEDICINE, *or, Family Adviser, consisting of Outlines of Anatomy, Physiology, and Hygiene, with Hints on the Practice of Physic, Surgery, and the Diseases of Women and Children, &c. &c.* By REYNELL COATES, M. D., Fellow of the College of Physicians of Philadelphia, &c., &c. Philadelphia: Carey, Lea, & Blanchard. 1838. 8vo. pp. 614.

THAT "the medical profession has opposed, at all times, the publication of works on domestic medicine," is Dr. Coates's very candid admission in his preface to the Popular Medicine. That, however, "the principal evils which have resulted, and are likely to result hereafter, from attempts at popular medical instruction, are attributable rather to the manner in which the subject has been treated, than to the nature of the subject itself," we are much disposed to doubt. The difficulty lies in the subject: medicine cannot be popularized. For the general ability with which he has fulfilled his task, Dr. Coates deserves high praise; to the excellence and practical value of the First Part of his work, the Outlines of Anatomy, Physiology, and Hygiene, we very readily bear testimony; but, we are compelled to add, that a perusal of the Practical Directions for the treatment of medical and surgical cases, although it has impressed us favorably of the style in which it is executed, has not satisfied us of the utility of such productions, nor shaken any of our objections to their publication. Popular treatises on medicine should be confined to the subjects of anatomy, physiology, and hygiene. As well physically as mentally, the proper study of mankind is man. Some acquaintance with the structure and with the healthful functions of the human body, should be considered essential parts of a liberal education. A knowledge, too, of the general principles of hygiene is within the scope of every intelligent mind, and cannot be too generally diffused. But within these boundaries lies the proper province of the popular writer on medicine. Within them he can be most useful, but he may not safely go beyond them. We cannot but regret, then, that Dr. Coates did not confine his labours to the production of a work like the Philosophy of Health of Dr. Southwood Smith; that he did not, indeed, stop short with the excellent first part of his book, nor venture into rivalry, however reluctantly, with the not very creditable host of "previous authors, with whom to affiliate," he tells us with a sincerity which we can understand, "falls not within the province of his ambition."

It is apparent from the tone of the author's introductory remarks, that he himself feels all the force of the general objections to the class of works in which his own must rank. We are, therefore, unwilling to urge them at any length. We would fain admit that there may be considerations of *expediency*, that justify his dereliction from what he admits to be *right in the abstract*. We are cheerfully disposed to give due weight to his plea, for a useful circulation of his book in growing Western populations, unsupplied with medical advice, and in Southern plantations, cut off from the rest of the world, and we shall heartily rejoice, if he can at all check the growth of the vile quackery, which is defiling the land.

One other point, and we have done. We must protest against the tender of the Popular Medicine as "a useful sketch to young men about commencing the study of medicine." The groundwork of a successful comprehension and application of therapeutics, is a knowledge of pathology and an aptitude at diagnosis, the results of long and painful previous training. The smattering, to be obtained from works like the Popular Medicine, can, at best, be but useless, and, by creating erroneous impressions, may do positive harm.

---

*The Louisville Journal of Medicine and Surgery.* Edited by Lunsford P. Yandell, M. D., Henry Miller, M. D., and Theodore S. Bell, M. D. Vol. I.—No. 1. Louisville, Ky.: Prentice & Weissinger. 1838. pp. 250. 8vo.

This is another evidence of the indomitable enterprise of the West, and we trust that it will be conducted in a spirit to merit and obtain adequate patronage to ensure its permanence. Its avowed connection with the Medical School of Louisville militates, we think, against that general independence and usefulness which should constitute the distinctive character of a scientific journal, and tends to encourage the idea that it may be made the organ of sectional feeling and prejudice. Nor, we must confess, are these doubts altogether removed by a perusal of the first number which is before us.

The contents are divided into Essays, Reviews, and Analecta. Of the original department, we cannot, with justice, speak in terms of unqualified approbation. The Original Communications are, in general, trite and uninteresting. The analytical notices are of works which have been for some time before the public, and upon whose merits they have long since decided. The selected department is meagre, and can lay no claims to novelty.

We have hazarded these remarks from no feelings of unkindness to the gentlemen who are embarked in the enterprise, whose motives we appreciate, and whose labors we sincerely wish to see prosper, but because we deem that an independent expression of sentiment is always demanded, at every sacrifice.

That the "Louisville Journal" may be eminently successful, and as useful as it is in the power of its able conductors to make it, is our very honest desire.

